# *DSP* missense variant in a Scottish Highland calf with congenital ichthyosis, alopecia, acantholysis of the tongue and corneal defects

**DOI:** 10.1186/s12917-021-03113-3

**Published:** 2022-01-07

**Authors:** Irene M. Häfliger, Caroline T. Koch, Astrid Michel, Silvia Rüfenacht, Mireille Meylan, Monika M. Welle, Cord Drögemüller

**Affiliations:** 1 Institute of Genetics, Vetsuisse Faculty, University of Bern, Bern, Switzerland; 2 Clinic for Ruminants, Vetsuisse Faculty, University of Bern, Bern, Switzerland; 3 Division of Clinical Dermatology, Vetsuisse Faculty, University of Bern, Bern, Switzerland; 4 Dermavet, Tierklinik Aarau-West, Oberentfelden, Switzerland; 5 Institute of Animal Pathology, Vetsuisse Faculty, University of Bern, Bern, Switzerland

**Keywords:** Cattle, Dermatology, Skin, Genodermatosis, Corneal ulcers, Hyperkeratosis, Genetic disorder, Precision medicine

## Abstract

**Background:**

Ichthyosis describes a localized or generalized hereditary cornification disorder caused by an impaired terminal keratinocyte differentiation resulting in excessive stratum corneum with the formation of more or less adherent scales. Ichthyosis affects humans and animals. Two rare bovine forms are reported, the severe harlequin ichthyosis and the less severe congenital ichthyosis, both characterized by a severe orthokeratotic lamellar hyperkeratosis.

**Results:**

A 2-weeks-old purebred Scottish Highland calf was referred because of a syndrome resembling congenital ichthyosis. The clinical phenotype included diffuse alopecia and a markedly lichenified skin covered with large and excessive scales. Additionally, conjunctivitis and ulceration of the cornea were noted. Post-mortem examination revealed deep fissures in the diffusely thickened tongue and histopathological findings in the skin confirmed the clinical diagnosis. Whole-genome sequencing of the affected calf and comparison of the data with control genomes was performed. A search for private variants in known candidate genes for skin phenotypes including genes related with erosive and hyperkeratotic lesions revealed a single homozygous protein-changing variant, *DSP*: c.6893 C>A, or p.Ala2298Asp. The variant is predicted to change a highly conserved residue in the C-terminal plakin domain of the desmoplakin protein, which represents a main intracellular component of desmosomes, important intercellular adhesion molecules in various tissues including epidermis. Sanger sequencing confirmed the variant was homozygous in the affected calf and heterozygous in both parents. Further genotyping of 257 Scottish Highland animals from Switzerland revealed an estimated allele frequency of 1.2%. The mutant allele was absent in more than 4800 controls from various other cattle breeds.

**Conclusions:**

This study represents the first report of combined lesions compatible with congenital ichthyosis, alopecia, acantholysis of the tongue and corneal defects associated with a *DSP* missense variant as the most likely underlying cause. To the best of our knowledge, this study is also the first report of a *DSP*-related syndromic form of congenital ichthyosis in domestic animals. The results of our study enable genetic testing to avoid the unintentional occurrence of further affected cattle. The findings were added to the Online Mendelian Inheritance in Animals (OMIA) database (OMIA 002243-9913).

**Supplementary Information:**

The online version contains supplementary material available at 10.1186/s12917-021-03113-3.

## Background

Ichthyoses encompass a heterogeneous group of congenital disorders characterized by an abnormal terminal keratinocyte differentiation [1]. They are linked by the common finding of a thickened stratum corneum resulting in localized or generalized scaling. In ichthyosis, desquamation of corneocytes is impaired, resulting in retained large squames and thus severe hyperkeratosis, as well as loss of skin elasticity and an abnormal barrier function [1, 2]. The thick skin with an excessive amount of superficial scales resembles fish scales (greek ichthys means fish) and gave the name for these disorders. In 2009, the various ichthyotic diseases in people were classified in a consensus paper [2]. In that milestone article, the classification is based on clinical findings and the inherited ichthyoses are subdivided into the large groups of syndromic versus non-syndromic forms. The further subdivision distinguishes between the common ichthyosis (ichthyosis vulgaris), autosomal recessive congenital ichthyosis, and keratinopathic ichthyosis [3]. The large group of recessively inherited disorders with congenitally appearing ichthyosis but no extra-cutaneous involvement is heterogeneous and can be subdivided in three clinical phenotypes: (1) Harlequin ichthyosis representing the most severe, mostly lethal phenotype; (2) congenital ichthyosis associated with erythema and fine white scales; and (3) lamellar ichthyosis characterized by large dark scales [3]. In humans, the various forms of ichthyosis are associated with variants in at least 50 genes encoding structural proteins and enzymes affecting several cellular functions including DNA repair, lipid biosynthesis, adhesion, desquamation, as well as other pathways [1–4].

In animals, several forms of non-syndromic presentations of ichthyosis have been reported and clinical signs are present at birth or rarely later in life [5]. A classification system similar to humans does not exist in animals, but localized and generalized forms are known and, based on histology, non-epidermolytic forms are distinguished from epidermolytic forms. Various forms of non-syndromic, mostly inherited forms of ichthyosis have been identified in domestic animals [5–12]. In cattle, forms of ichthyosis fetalis, which represents a severe form also described as harlequin-like ichthyosis (OMIA 002238-9913) and congenital ichthyosis (OMIA 001993-9913) have been reported. In older reports, the diagnosis was based only on the clinical and/or histopathological findings. In cattle, affected animals show variable degrees of generalized hyperkeratosis; large thick cutaneous scales situated mainly to the inguinal region, limbs, abdomen and muzzle are typical [8, 9]. In some syndromic cases, microtia, cataracts and thyroid abnormalities have been reported as well [12]. Calves affected with congenital ichthyosis show milder but comparable lesions to those of ichthyosis fetalis [10]. Changes in the hair coat such as hypotrichosis or alopecia are clinically described but may represent secondary findings.

The underlying genetic causes for ichthyosis fetalis are mostly unknown in cattle, except for an *ABCA12*-related harlequin-like form (; OMIA 002238-9913). This recessively inherited lethal disorder observed in Chianina cattle is characterized by a hyperkeratotic and significantly lichenified skin with widely distributed, erytematous, deep fissures [7, 8, 13]. Two further pathogenic loss-of-function variants in the bovine *ABCA12* gene associated with nonviable forms of ichthyosis fetalis have been reported in Shorthorn [14] and Hereford cattle [15]. In addition, a deleterious frameshift variant in the *FA2H* gene of Chianina cattle associated with a mild syndromic form of bovine ichthyosis congenita (OMIA 002450-9913) has recently been reported [16].

At present, this obvious genetic heterogeneity of inherited cornification disorders can be analyzed in cattle using whole-genome sequencing (WGS)-based precision diagnostics [17]. Studying the molecular aetiology of single cases is nowadays also feasible in cattle [18–20]. Therefore, the purpose of this study was to characterize the clinical and pathological phenotype of an ichthyosis-affected Scottish Highland calf, and to evaluate its possible genetic etiology using WGS.

## Methods

WGS was performed on DNA extracted from ethylenediaminetetraacetic acid (EDTA) blood of the calf. An individual PCR-free fragment library with approximately 400 bp inserts was created and sequenced on a NovaSeq6000 for 150 bp paired-end reads (Illumina, San Diego, CA, USA). The sequenced reads were mapped to the ARS-UCD1.2 reference genome [21] resulting in an average read depth of approximately 19-fold, and single-nucleotide variants and small indel variants were called in accordance as described before [22]. The applied software and steps to process fastq files into binary alignment map and genomic variant call format files were in accordance with the processing guidelines of the 1000 Bull Genomes Project [23]. The effects of the called variants were functionally evaluated with snpeff v4.3 [24], using the NCBI annotation release 106 (https://www.ncbi.nlm.nih.gov/genome/annotation_euk/Bos_taurus/106/). In order to find private variants, we compared the genotypes of the affected calf with 705 cattle genomes of various breeds that had been sequenced in the course of the Swiss Comparative Bovine Resequencing project [25]. An *in silico* assessment of the molecular consequences of the identified amino acid exchanges was carried out with PROVEAN [26].

## Results

### Clinicopathological findings

**Fig. 1:**
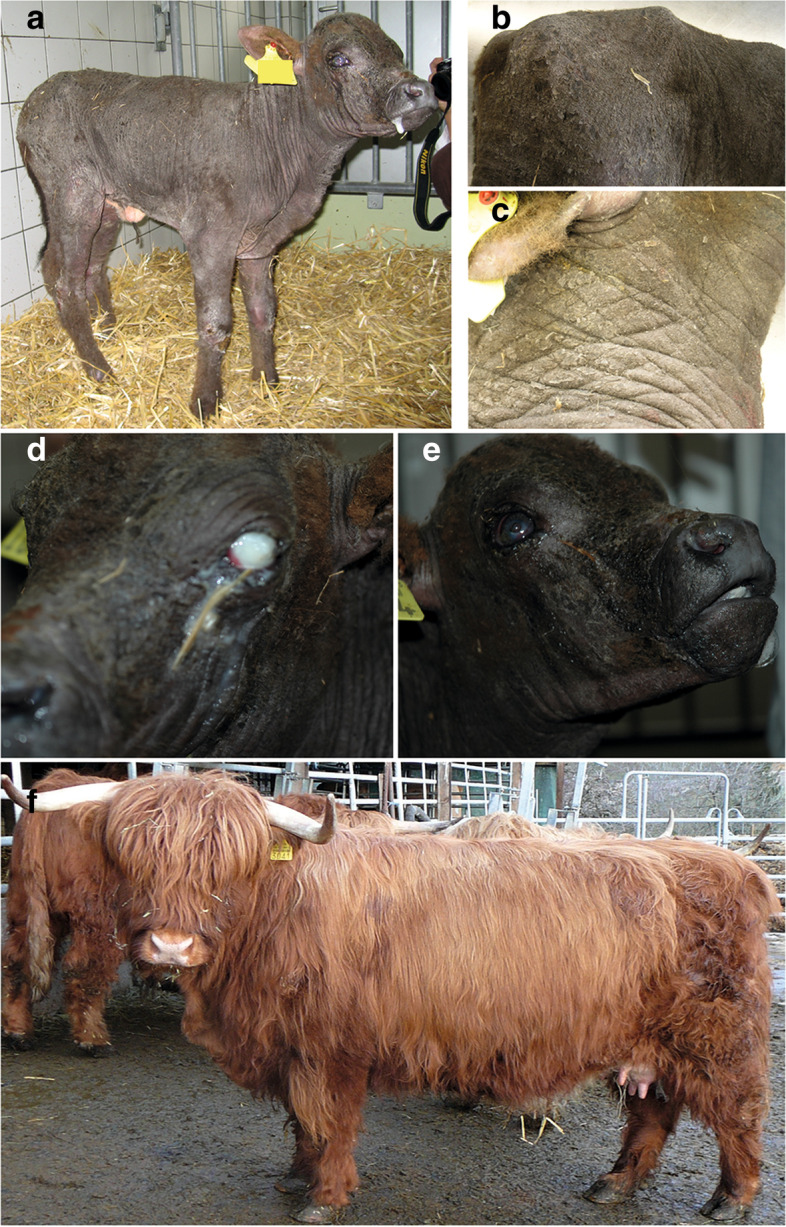
Clinical phenotype of a 2-week-old Scottish Highland calf with congenital ichthyosis, alopecia and corneal defects. Note the flat crusts lateral to the tarsi, dorsal to the metacarpi and on the bride of the nose (**a**). Backside of the calf with alopecia and large scales (**b**). Alopecia and lichenification on the neck (**c**). Cloudy left eye with conjunctivitis (**d**). The right eye shows a corneal ulceration with a prolapsed iris (**e**). Appearance of a typical purebred Scottish Highland cow (**f**)

A 2-week-old female Highland cattle calf weighing 51 kg was presented to the Clinic for Ruminants of the Vetsuisse Faculty of the University of Bern with severe skin lesions (Fig. 1). The calf presented with diffuse alopecia involving approximately 90% of the body. The remaining hair was found on the head, the limbs and the tail, and was easy to pull. The markedly lichenified hyperkeratotic skin was covered by a thick layer of keratin, which exfoliated as large scales (Fig. 1c). The skin presented multiple folds (Fig. 1a). In addition, the areas lateral to the tarsi, dorsal to the metacarpi and the bridge of the nose were covered with flat crusts (Fig. 1a). The eyelids showed hyperkeratosis as well (Fig. 1d). The ears were of normal length and the mucocutaneous junctions were unaltered (Fig. 1a). The epithelium on the dorsal surface of the tongue was diffusely thickened and presented with multifocal fissures (Additional file 1: Fig. S1). Necrotic debris was covering the surface of the tongue multifocally. There were whitish plaque-like deposits on the ventral tip of the tongue (Fig. 1d). The hoofs seemed intact.

**Fig. 2:**
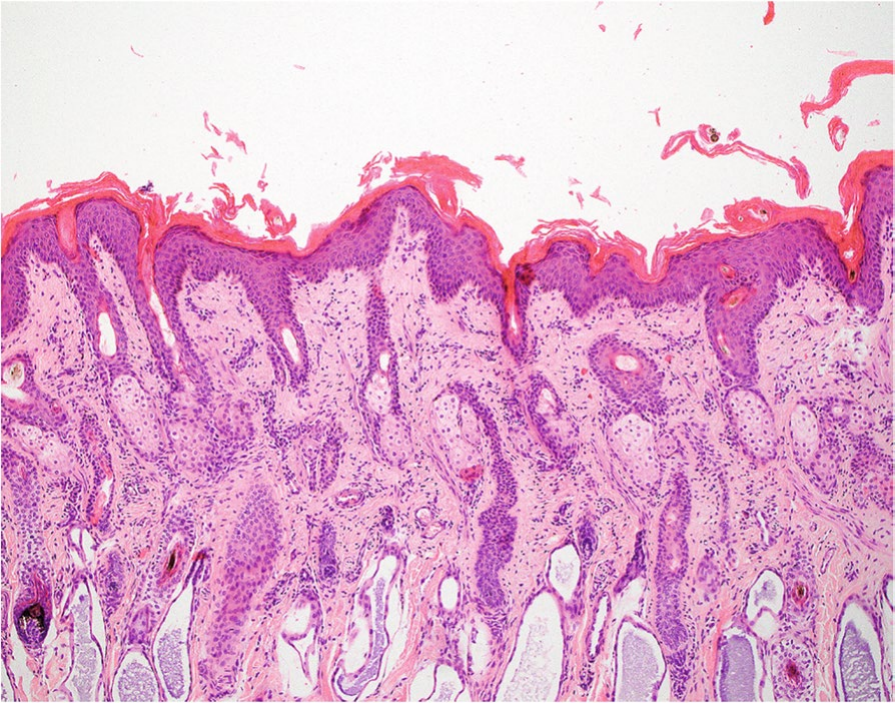
Histological investigation of the skin of a 2-week-old Scottish Highland calf with congenital ichthyosis and alopecia. Note the compact to laminated orthokeratotic hyperkeratosis extending into the follicular infundibuli and the mild to moderate hyperplasia of the epidermis. Haematoxylin and eosin stain, x100

Due to the severity of the skin lesions and the poor prognosis, the calf was euthanized one day after admission and necropsy with subsequent histological examination of various tissues including the skin and the tongue was performed. Histologically, the epidermis was mildly to moderately hyperplastic and covered with abundant compact to laminated keratin and multifocal crusts (Fig. 2). The hyperkeratosis extended into the follicular infundibulum and sometimes into the ducts of the sebaceous glands. Many hair follicles were dysplastic, presenting with a false orientation of the infundibula and isthmic part, a distorsion of the inferior portion, a multifocally thickened outer root sheath and hair shafts, which were either broken or had an irregular contour. In addition, there was a mild perivascular infiltrate composed mostly of lymphocytes and plasma cells in the superficial dermis.

**Fig. 3:**
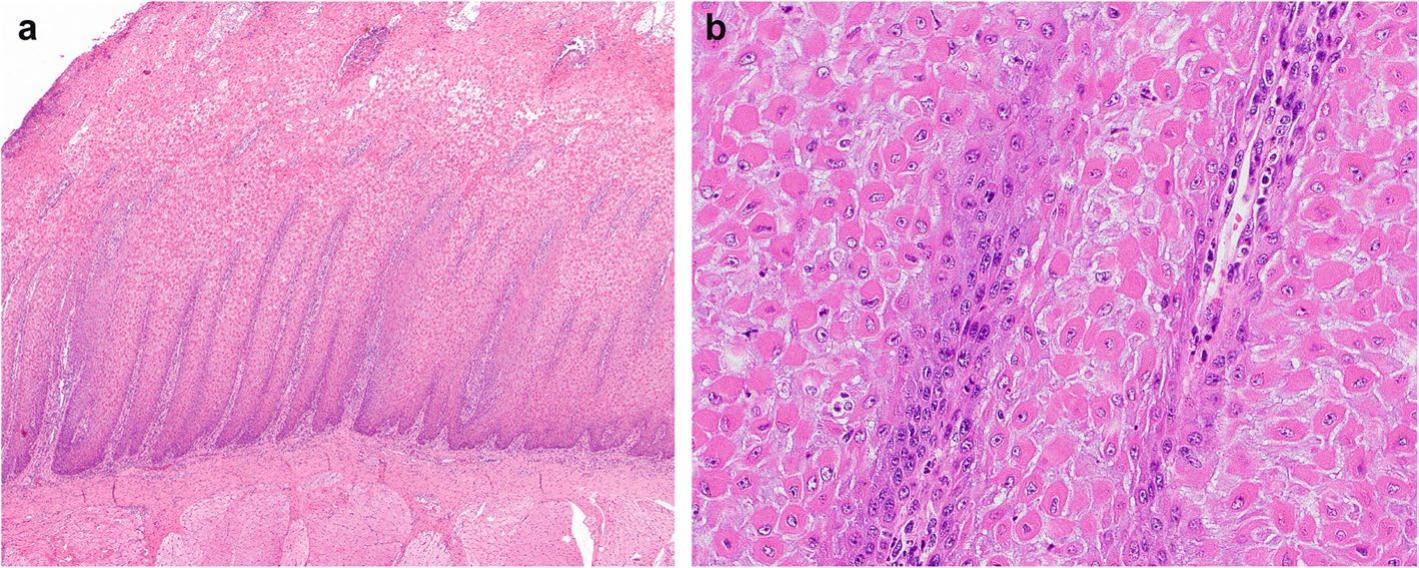
Histological investigation of the tongue of a two-week-old Scottish Highland calf with congenital acantholysis of the tongue. Note the severely hyperplastic epithelium and the rounded keratinocytes losing the adhesion to the neighbouring cells (**a**) as well as the mild infiltration of lymphocytes and plasma cells in the lamina propria (**b**). Hematoxylin and eosin stain, x100 (**a**), x400 (**b**)

The epithelium of the tongue was diffusely hyperplastic and presented with multifocal deep fissures (Fig. 3a). The keratinocytes in the stratum spinosum of the tongue epithelium were rounded and towards the surface adhesion to the neighboring keratinocytes was completely lost (acantholysis). Necrotic debris was present on the surface. On the tongue, the mucosa of the dorsal surface showed a marked parakeratotic hyperkeratosis (Fig. 3a). A mild infiltration of lymphocytes and plasma cells in the lamina propria was seen as well (Fig. 3b). The fissures presented histologically as full thickness ulceration and serocellular crust formation.

The cornea of the right eye presented with a focal perforating ulcer of 5 mm in diameter with a partial prolapse of the iris through the ulcerated surface and attachment of the iris to the cornea (Fig. 1d). Histologically the cornea neighboring the transmural ulcer was infiltrated with lymphocytes and neutrophils. Bacteria were also present. In addition, new vessel formation in the cornea was seen. The iridocorneal angle was constricted and contained many neutrophils. The retina was detached. The left eye was macroscopically cloudy and conjunctivitis was present (Fig. 1e). Histopathological examination of the left eye revealed a transmural ulceration of the cornea and rupture of the Descemet’s membrane. Large amounts of fibrin, neutrophils and bacteria were found in the anterior chamber. The iris was attached to the cornea and the retina was diffusely ablated.

Besides these major pathological findings, a mild acute purulent bronchopneumonia and a diffuse severe subacute pustular rumenitis without hyperkeratosis were noted. Other tissues were unremarkable. In summary, the clinical and pathological findings in the skin of this calf were consistent with congenital ichthyosis and a follicular / hair shaft dysplasia. The findings in the tongue and the eyes are remarkable and have, to the best of our knowledge, not been described in association with similar skin lesions.

### Pedigree analysis

**Fig. 4:**
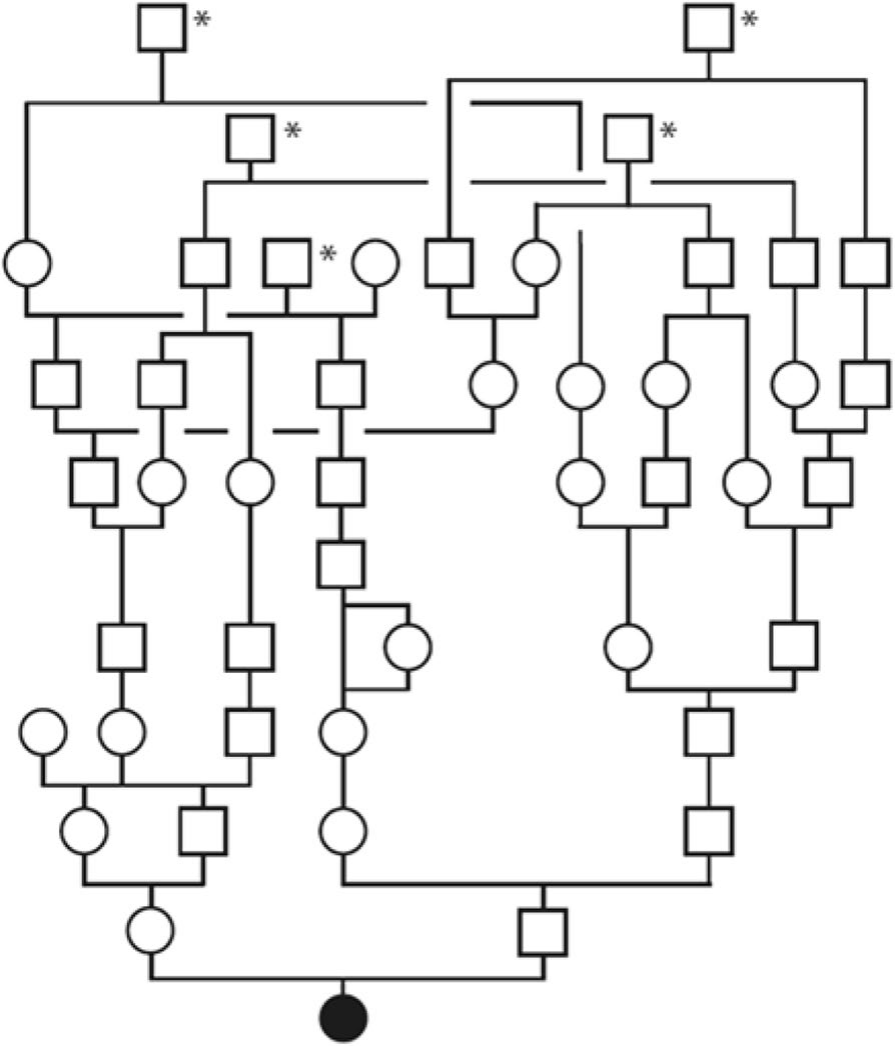
Pedigree of the Scottish Highland calf with a syndromic form of congenital ichthyosis. Note the multiple inbreeding loops between the parents. Five common ancestors are marked by an asterisk. The affected calf is marked with a black circle. Females are indicated as circles and males as squares

The studied calf was the only affected animal in a Swiss herd of purebred Scottish Highland cattle. The sire of the present case was a natural service purebred Scottish Highland bull, which sired further 105 normal offspring within eight years. An enquiry with the Scottish Highland cattle breeders in Switzerland revealed no evidence of other similar cases in the past. The available pedigree records of the calf’s ancestry were analyzed and multiple inbreeding loops between the parents were found (Fig. 4). We detected at least five common ancestors occurring 7-8 generations ago. In light of the obvious consanguinity as well as both parents are unaffected, we hypothesized that the current case might be explained by a rare recessively inherited variant. Nonetheless, due to the sporadic occurrence a *de novo* mutation in the germline of one parent or during very early embryonic development could not be fully excluded.

### Genetic findings

Based on the assumed recessive inheritance and a sub-lethal effect of the disease-causing variant, we hypothesized that, most likely, a loss-of-function mutation affecting a protein-coding gene would be responsible for the observed disorder. Therefore, we subsequently concentrated on protein-changing variants with a moderate or high predicted impact on the encoded protein. This revealed five protein-changing variants, located within different genes or loci, exclusively present homozygous in the genome of the ichthyosis-affected calf (Additional file 2: Table S1). Of all these five remaining private variants, only one occurred in a candidate for ichthyosis: desmoplakin (*DSP*). The variant can be designated as Chr23:47826600G>T. It is a missense variant, NM_001192368.2:c.6893 C>A, predicted to change a highly conserved alanine residue at the C-terminal plakin domain affecting the second plakin-repeat subdomain of desmoplakin, NP_001179297.1:p.(Ala2298Asp). The *DSP* missense variant was predicted to be deleterious (Table S1).

**Fig. 5:**
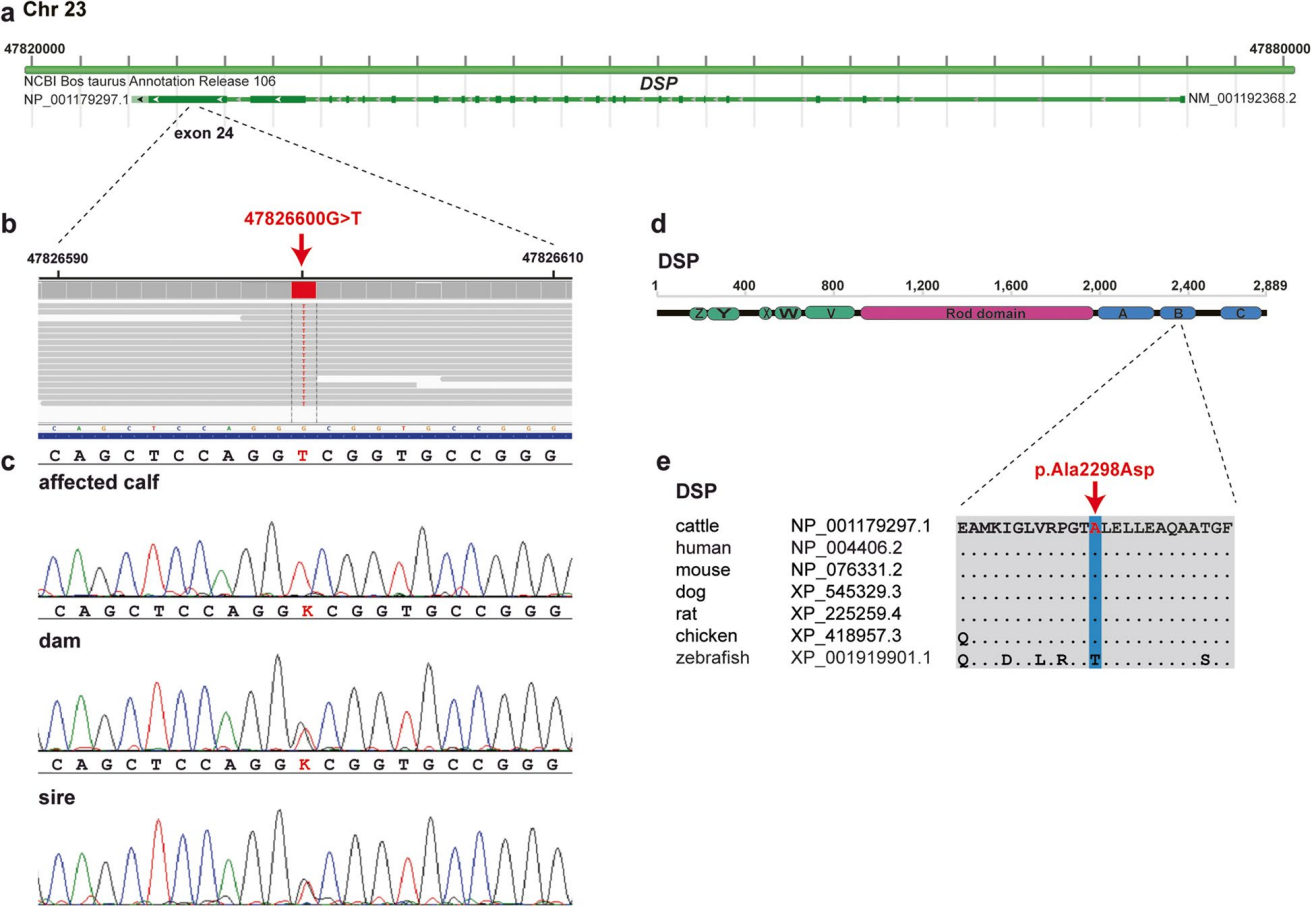
A missense variant in the *desmoplakin* (*DSP*) gene is associated with a syndromic form of congenital ichthyosis in Scottish Highland cattle. Schematic diagram of the *DSP* gene showing the location of the candidate pathogenic variant in exon 24 (**a**). Screenshot of the WGS data shows the homozygous single nucleotide variant (red arrow) present in the affected calf (**b**). Electropherograms from Sanger sequencing data (**c**). Note that the DNA sample from the affected calf carried the mutant allele in a homozygous state, while the parents were heterozygous, as expected for obligate carriers. The bovine desmoplakin protein is composed of the N-terminal plakin domain with 5 α-helical bundles (Z, Y, X, W, V), the coiled-coiled rod domain, and the C-terminal plakin domain with the 3 plakin-repeat subdomains (A, B, C) (**d**). Multiple-species DSP protein alignment (**e**). The predicted change of the highly conserved alanine to aspartic acid in the affected calf is highlighted by a red arrow

This variant affecting a candidate gene for loss of keratinocyte adhesion explains the acantholysis in the tongue of the calf well. It may also affect the corneodesmosomes in the epidermis and is likely the pathogenic variant for the observed phenotype. We confirmed the presence of the *DSP* missense variant by Sanger sequencing (Fig. 5). The mutant *DSP* allele showed the expected co-segregation with ichthyosis in the available family trio, this was not the case for the other four protein-changing variants in which both parents also showed the alternative homozygous genotype (Table S1). Genotyping of 257 Scottish Highland cattle revealed no homozygous mutant animal and a total of six heterozygous carriers including both parents confirming recessive inheritance. Furthermore, the variant was absent in 4109 cattle genomes of a variety of global breeds including 12 Scottish Highland animals that were part of the run 8 of the ongoing 1000 Bull Genomes Project [23].

## Discussion

The aim of this study was to characterize the clinicopathological phenotype and the genetic aetiology of the observed lesions in the skin, the tongue and the eyes of a Highland calf. The skin lesions are compatible with congenital ichthyosis, which has never been reported in a purebred Scottish Highland calf. Herein we also present evidence for the occurrence of a novel form of recessively inherited ichthyosis due to a homozygous missense variant in the bovine *DSP* gene, which enables selection against this disorder. We hypothesize that this most likely pathogenic deleterious variant is also causative for the alopecia, the erosive tongue lesions due to severe acantholysis and the corneal ulcers. Due to the consanguinity and in light of the syndromic disease phenotype, there is theoretically the possibility of a second genetic disorder; however, the genome sequencing carried out did not reveal any evidence of a second pathogenic protein-changing variant.

Desmoplakin is part of all desmosomes, which are abundantly expressed in both myocardial and epidermal tissue and serve as intercellular adhesion molecules to resist mechanical stress. It anchors intermediate filaments to desmosomal plaques and forms an obligate component of functional desmosomes. Mutations in genes encoding for desmosomal components are associated with a broad spectrum of phenotypes comprising skin and hair abnormalities, and account for 45-50% of cases of arrhythmogenic right ventricular cardiomyopathy in humans [27]. More than 120 dominant and recessive *DSP* variants have been reported to be associated with skin, hair and/or heart defects such as dominant inherited arrhythmogenic right ventricular cardiomyopathy (OMIM 615,821) and recessive inherited Carvajal syndrome (OMIM 605,676) characterized by an extreme type of dominant arrhythmogenic right ventricular cardiomyopathy/dysplasia associated with woolly hair and epidermolytic palmoplantar keratoderma. The skin fragility-woolly hair syndrome (OMIM 607,655) represents another recessive disorder due to desmoplakin mutations characterized by palmoplantar keratoderma, woolly hair, variable alopecia, dystrophic nails, and excessive blistering. In these cases, as well as in the bovine case described here, there is no cardiomyopathy. Lethal acantholytic epidermolysis bullosa (OMIM 609,638), characterized by severe blistering of the epidermis and mucous membranes, is caused by homozygous deletions causing truncation of the DP tail [28]. Heterozygous carriers of any of these known recessive mutations displayed no phenotypic abnormalities in humans [29]. In general, epidermal fragility or excessive cornification is the first manifestation of these *DSP*-related human diseases, the hair changes (woolly hair or hypotrichosis) as well as the palmoplantar keratosis appear during childhood. The combination of these clinical features is an alarm sign for cardiomyopathy, which can appear at a young age in the form of severe arrhythmias, heart failure or spontaneous cardiac death [30]. In the *DSP*-associated case in cattle presented here, hyperkeratosis of the skin, changes in the hair coat as well as disorders of the mucous membranes are present. Therefore, at first glance, it resembles more the autosomal recessive forms of lethal acantholytic epidermolysis bullosa and/or skin fragility woolly hair syndrome than other *DSP*-related human diseases. Nevertheless, clear differences in manifestation are visible in detail, e.g. no evidence of blistering in the epidermis. Furthermore, there were no signs of heart problems, but a very young animal was examined and it cannot be ruled out that such changes would have occurred later in life. Also in human the classification of DSP-related phenotypes and the genotype-phenotype correlations are challenging, partly because different terms are used to designate disorders that comprise similar clinical features in the literature [306]. We therefore suspect that the specific *DSP* missense variant, which we identified here for the first time in a mammalian species, causes a highly probable unique syndromic disease phenotype. In most human cases, the precise consequences of the variants and the molecular pathology remain elusive due to the lack of expression and functional studies.

An exactly corresponding variant affecting human DSP residue 2288 was not yet described, and it not known as a variant in comprehensive databases such as gnomAD [31]. Interestingly, in a case report of a 14-year-old child with extensive mucocutaneous blisters caused by acantholysis of keratinocytes, epidermolytic palmoplantar keratoderma, nail dystrophy, enamel dysplasia, and sparse woolly hair, a very similar *DSP* missense variant was found [32]. This pathogenic variant results in a substitution of an aspartic acid for a conserved alanine residue at amino acid 2655 located in the C-terminal plakin domain of desmoplakin, affecting the third plakin-repeat subdomain of the human desmoplakin protein. The three tandem plakin repeat regions in the C-terminus of desmoplakin mediate binding to intermediate filaments. Its association with epidermal and simple keratins is dependent on the tertiary structure induced by heterodimerization of these intermediate filament proteins. Similar to the non-conservative amino acid replacement in the herein presented calf with acantholysis of the tongue epithelium, impaired desquamation of corneocytes in the skin (ichthyosis congenita), follicular / hair shaft dysplasia and corneal ulcers, such an exchange of a nonpolar, hydrophobic alanine with a charged acidic aspartate is most likely causative. In the previously cited human case description, it was reported that, although the variant does not significantly alter the three-dimensional structure of desmoplakin, structural modelling indicates changes in the electrostatic potential of the affected protein region [32]. The authors speculated that the change may seem subtle but the clinical phenotypes suggest that it alters intermediate filament binding functions that depend on electrostatically driven intermolecular interactions. Interestingly, immunofluorescence microscopy showed a reduction in the C-terminal domain of desmoplakin in the skin and oral mucosa of the child carrying this missense variant. Therefore, we conclude that the missense variant identified in the affected Scottish Highland calf represents a plausible candidate causative mutation for the lesions observed in this calf in the skin, the hair follicles, the tongue and the eyes. It is possible that the expression of the affected protein differs between the epithelium of the tongue and the epidermis, resulting in acantholysis in the tongue and impaired corneocyte desquamation in the skin. Impaired desmosome adhesion between the trichocytes of the hair shaft may also explain the alopecia and is supported by the histologically observed hair shaft dysplasia. Finally, the severe abnormalities of the cornea in the affected calf might be explained by the impaired function of desmoplakin as the corneal epithelium expresses a subgroup of keratins similar to those of epidermal epithelium [33]. Human genodermatoses, often have extracutaneous manifestations, and ocular manifestations in particular can have significant clinical implications, such as blindness [34]. The skin and eye malformations found in the affected calf resemble human keratitis-ichthyosis-deafness syndrome (OMIM 148,210), a rare disorder caused by dominant acting variants in *GJB2* that encodes for connexin 26, a gap junction protein.

## Conclusions

Rare disorders in livestock animals are traditionally poorly diagnosed. The report of this single case by a concerned breeder, followed by the diagnosis of ichthyosis congenita, follicular dysplasia, acantholysis of tongue epithelium, severe cornea defects, in combination with WGS has resulted in the identification of a most likely pathogenic variant in the *DSP* gene. This report should alert breeders of Scottish Highland cattle about the possible emergence of congenital ichthyosis in the future and will permit genetic testing to avoid the unintentional occurrence of further affected cattle. Screening the variant in the global Scottish Highland cattle population will enable better assessment of the population allele frequency for this breed-specific deleterious variant. Future studies to assess the functionality of the DSP protein in the presence of the missense variant will be valuable for understanding the biological impact of the mutation. To the best of our knowledge, this study represents the first report of a *DSP*-related syndromic form of congenital ichthyosis in domestic animals. The observed acantholysis of the tongue and corneal defects add *DSP* to the list of candidate genes for similar congenital phenotypes in humans.

## Supplementary Information

**Additional file 1: Figure S1.** Macroscopic pictures of the tongue of the affected calf after necropsy.

**Additional file 2: Table S1.** Private homozygous coding variants in the sequenced case. List of the remaining variants after the comparison to the control cohort of 705 genomes of other breeds, revealing 5 protein-changing variants only present in the genome of the ichthyosis-affected calf. These 5 variants with a moderate or high predicted impact on the encoded protein were located within 5 different genes or loci. Note that the predicted pathogenic variant NM_001192368.2: c.6893 C>A is the only one located in a functional candidate gene

## Data Availability

The WGS data of the case can be found in the European Nucleotide Archive under the sample accession no. SAMEA5714970.

## Abbreviations

DSP: Desmoplakin
OMIA: Online Mendelian Inheritance in Animals
OMIM: Online Mendelian Inheritance in Man
WGS: Whole-genome sequencing
