# Supplementary figures and images for: DSP missense variant in a Scottish Highland calf with congenital ichthyosis, alopecia, acantholysis of the tongue and corneal defects
Source: BMC Vet Res. 2022 Jan 7;18:20. doi: 10.1186/s12917-021-03113-3 (PMC8739657; doi:10.1186/s12917-021-03113-3)

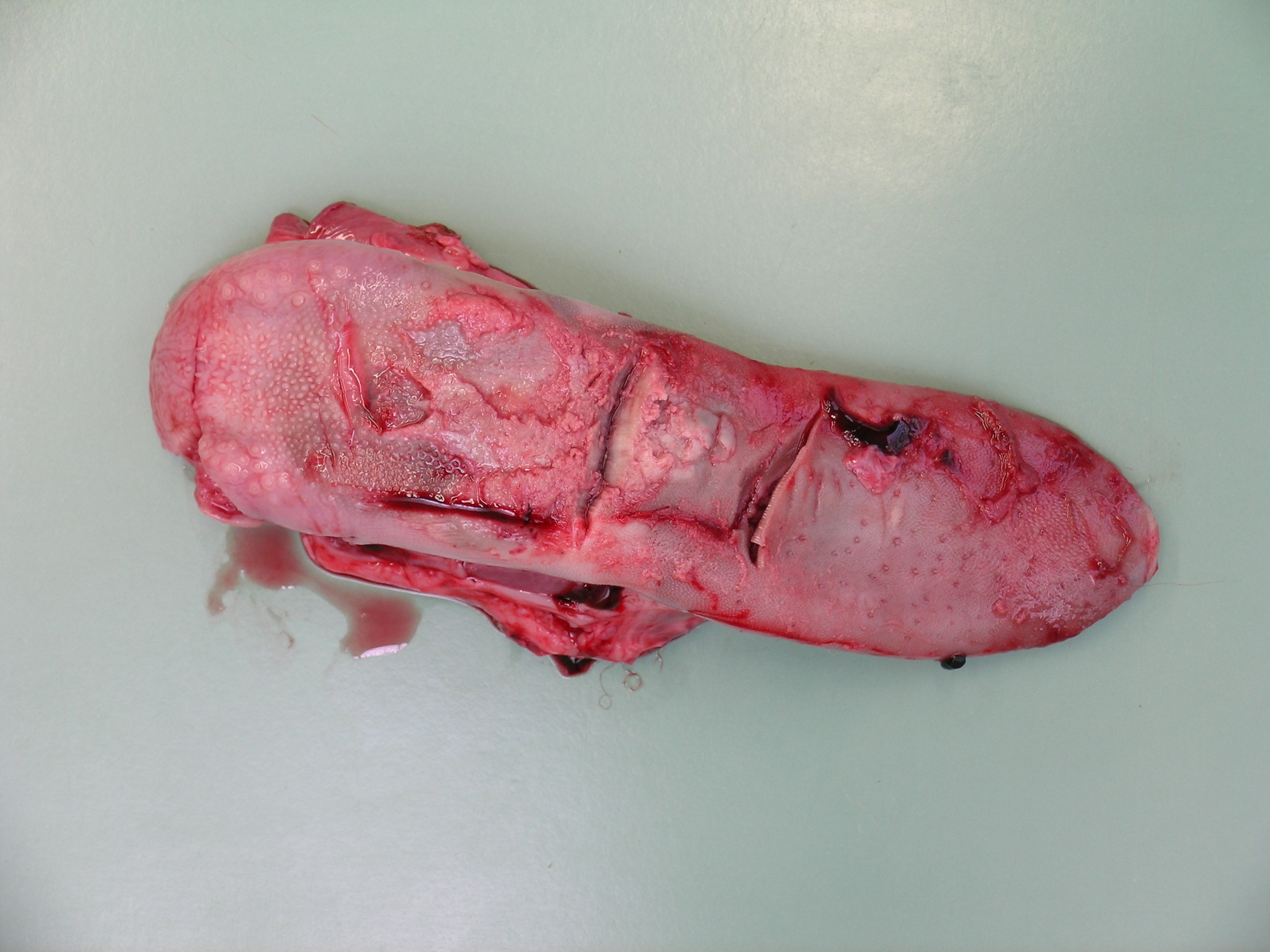

Supplement: Supplementary file 1 — Additional file 1: Figure S1. Macroscopic pictures of the tongue of the affected calf after necropsy. [file 12917_2021_3113_MOESM1_ESM.jpg]
